# High-Frequency Repetitive Transcranial Magnetic Stimulation Over the Left Dorsolateral Prefrontal Cortex Shortly Alleviates Fatigue in Patients With Multiple System Atrophy: A Randomized Controlled Trial

**DOI:** 10.3389/fneur.2021.755352

**Published:** 2022-01-11

**Authors:** Jing Pan, Tao-Mian Mi, Jing-Hong Ma, Hong Sun, Piu Chan

**Affiliations:** ^1^Department of Neurology, Neurobiology and Geriatrics, Xuanwu Hospital of Capital Medical University, Beijing Institute of Geriatrics, Beijing, China; ^2^Department of Neurology, The Second Affiliated Hospital of Inner Mongolia Medical University, Hohhot, China; ^3^Clinical Center for Parkinson's Disease, Key Laboratory for Neurodegenerative Disease of the Ministry of Education, Beijing Key Laboratory for Parkinson's Disease, Parkinson's Disease Center of Beijing Institute of Brain Disorders, Collaborative Innovation Center for Brain Disorders, XuanWu Hospital of Capital Medical University, Beijing, China; ^4^National Clinical Research Center for Geriatric Disorders, Xuanwu Hospital of Capital Medical University, Beijing, China

**Keywords:** transcranial magnetic stimulation, the left dorsolateral prefrontal cortex, multiple system atrophy, fatigue, effective

## Abstract

**Background:** Fatigue is a common symptom in patients with Multiple system atrophy (MSA), but effective treatments remain elusive. The present study aims to investigate whether high-frequency repetitive transcranial magnetic stimulation (rTMS) over the left dorsolateral prefrontal cortex (DLPFC) could relieve fatigue in patients with MSA.

**Methods:** This is a single-center, randomized and double-blind trial. Twenty-two patients with MSA and fatigue were randomly allocated to receive 10 sessions of either active (*N* = 11) or sham (*N* = 11) 10 Hz rTMS over the left DLPFC. The participants were assessed at baseline (T0), after the last session of treatment (T1), and at 2-week (T2), and 4-week (T3) follow-up timepoints. The primary outcomes were Fatigue Severity Scale-9 (FSS-9) scores, with Unified Multiple System Atrophy Rating Scale (UMSARS), 17-item Hamilton Depression Scale (HAMD-17), and Hamilton Anxiety Scale (HAMA) as secondary outcomes.

**Results:** Two-way repeated ANOVAs revealed significant group × time interactions for FSS-9 scores (*p* < 0.001), HAMD-17 scores (*p* = 0.01), HAMA scores (*p* = 0.01), and UMRSA part II (*p* = 0.05). *Post-hoc* analyses showed that compared to T0, the active group exhibited remarkable improvements in FSS-9 and UMRSA part II scores at T1 and T2, but not at T3, and also in HAMD-17 and HAMA scores at T1, T2, and T3. No significant improvement was found in the sham group.

**Conclusion:** High-frequency rTMS over the left DLPFC could provide short-term improvements for alleviating fatigue in patients with MSA, but the beneficial effects last no more than 4 weeks.

## Introduction

Multiple system atrophy (MSA), an orphan, adult-onset, sporadic, progressive neurodegenerative disease, is characterized by Parkinsonian features, cerebellar ataxia, and autonomic failure in various combinations (1). Early and severe autonomic failure is a core feature of MSA, including fatigue (2). Studies have shown that fatigue, an independent non-motor symptom, is one of the most common and major problems for 38–61% MSA patients, which contributes greatly to reduced social participation and quality of life (3–5). Treatment of fatigue can be very challenging, as till now there is no widely accepted treatment protocol available, including pharmacological treatment, deep brain stimulation, and rehabilitation strategies (5, 6).

Repetitive transcranial magnetic stimulation (rTMS), a potent tool and non-invasive means of electrically stimulating neurons in the cerebral cortex, is able to modify neuronal activity of targeted cortical areas and has been widely applied to treat various neurological conditions (7). Several studies have shown that rTMS could alleviate the severity of motor disability in MSA patients (8–10). To our best knowledge, however, no study has so far specifically investigated the effects of rTMS on fatigue in patients with MSA. Previous studies have demonstrated that rTMS over the left dorsolateral prefrontal cortex (DLPFC) help improve fatigue symptom in some other neurological disorders, including fibromyalgia, myalgic encephalomyelitis, and multiple sclerosis (11–14). Moreover, high-frequency rTMS with an optimal frequency of 10 Hz applied to the DLPFC has been suggested as a potent treatment for fatigue with a Level A evidence (11–14). Here, in the present study, we aimed to investigate the effect of high-frequency rTMS over the left DLPFC on fatigue in patients with MSA. We hypothesized that high-frequency rTMS over the left DLPFC can alleviate fatigue in patients with MSA.

## Materials and Methods

### Participants

Twenty-two MSA patients with fatigue were eligible for the study from the Movement Disorders Center of the Xuanwu Hospital of Capital Medical University in Beijing, China. Patients were diagnosed as possible or probable MSA according to the second consensus statement on the diagnosis of MSA (2). Inclusion criteria were: (a) 30–75 years old, (b) Presence of clinical fatigue: Fatigue Severity Scale-9 (FSS) ≥36, and (c) stable anti-Parkinsonian therapy for ≥4 weeks and constant medication regimens throughout the trial. Exclusion criteria included: (a) Mini-Mental State Examination scores (MMSE) ≤ 24, (b) presence of contraindications for rTMS. The study protocol was approved and supervised by the Xuanwu Hospital Ethics Committee; all patients had agreed and confirmed informed consent prior to the study. The present study was registered at the Clinical Trial Registration (http://www.clinicaltrials.gov, NCT 04313530).

### Experimental Design

This study was a single-center, randomized, double-blind, and sham-controlled trial, in which the 22 participants were randomly assigned (with 1:1 ratio) with sealed envelopes into two groups to receive either 10-Hz rTMS (*N* = 11) or sham stimulation (*N* = 11) over the left DLPFC. Both the participants and researchers were blind to the randomization group, only the clinician responsible for the rTMS protocols was unmasked to the randomization sequence.

### rTMS and Sham Protocols

Magnetic stimulation was applied using a 7-cm, handheld, figure-of-eight coil was connected to a biphasic magnetic stimulator (Magstim Rapid; TheMagstim Co. Ltd., UK). The treatment protocol was performed in a total of 10 sessions over two successive weeks, consisting of one session per day for five consecutive days followed by a 1-day interval. Intervention was given at approximately the same time of day for each participant. The rTMS parameters used in the present study were referred to several previous studies, which have reported beneficial effects of rTMS on fatigue (11–14). That is, each session consisted of 20 series of 2-s 10 Hz pulses followed by a 18 s interval, with an intensity of 100% resting motor threshold (RMT), which gave a total of 1,200 pulses per session. The RMT is defined as the minimum intensity to evoke a visible voluntary contraction of the target muscle, the thenar muscles of the right hand, in 50% of successive trials. The coil was oriented at a 45° angle to the midsagittal plane (15) and was fixed to an arm that could be adjusted in three dimensions. Sham stimulation protocol was same as the rTMS protocol, except the coil was oriented at a 90° angle to the midsagittal plane (15), which could produce similar sounds and sensations as active stimulation while not inducing currents within the brain. All the participants were arranged at different time to avoid them from discussing with each other which ensured blinding during the data collection process.

### Clinical Assessments

The participants underwent clinical assessments at baseline (T0), and three follow-up timepoints, that is, immediately after the tenth treatment session (T1), 2 weeks (T2), and 4 weeks (T3) after T1. The primary outcome was the FSS score, a self-reported scale for assessing the fatigue severity over the last 2 weeks. The secondary outcomes were the part I and II of the Unified Multiple System Atrophy Rating Scale (UMSARS), the Hamilton Depression Scale (HAMD), and the Hamilton Anxiety Scale (HAMA), which were used to assess motor performance and non-motor symptoms, respectively.

### Side Effects

The safety of rTMS was assessed by monitoring the occurrence of adverse effects for all patients during the whole study process. These side effects were recorded at the T0, T1, T2, and T3 timepoints and were grouped into the following categories: (1) headache, (2) site discomfort, (3) nausea, (4) dizziness, and (5) others.

### Statistical Analysis

All statistical analyses were performed using SPSS Version 26 (IBM, Chicago, IL, USA). Demographic data were presented as mean ± SD for continuous variables and ratios or percentages for categorical variable. Independent two samples *t*-test was used to compare continuous variables, and the χ^2^-test was performed for the comparison of categorical variables. Two-way repeated ANOVA, with Group (rTMS/sham group) as between-subject factor and Time (T0, T1, T2, T3) as within-subject factor, was applied to estimate the effects of rTMS on the clinical outcomes. The threshold for the level of significance was set at α = 0.05. In all cases, *P*-values < 0.05 was considered to defined as statistically significant result.

## Results

### Participants

The demographic and clinical characteristics of the participants are presented in Table 1. The rTMS and sham group had similar baseline characteristics including age, gender, H-Y stage, levodopa-equivalent daily dose (LEDD), UMSARS scores, HAMA, HAMD, GDS, MMSE, and MoCA. The severity of fatigue was basically the same between the two groups.

**Table 1:** Demographic and clinical features of participants.

**Variables**	**rTMS group**	**Sham group**	***P***
	**(*N* = 11)**	**(*N* = 11)**	
Gender (female/male)	6/5	6/5	1.00
Age (years)	58.64 ± 5.50	59.00 ± 6.02	0.73
Disease duration (years)	2.00 ± 1.00	1.91 ± 1.22	0.35
Subtypes (MSA-P/MSA-C)	6/5	4/7	1.00
H-Y stage	2.95 ± 1.19	2.86 ± 1.23	0.44
UMSARS I	18.45 ± 8.03	22.45 ± 6.76	0.99
UMSARS II	18.82 ± 9.31	20.27 ± 8.01	0.91
UMSARS IV	2.18 ± 1.25	2.72 ± 1.27	0.48
LEDD (mg/d)	295.45 ± 313.41	234.09 ± 295.80	0.37
MMSE	28.64 ± 1.86	27.64 ± 1.57	0.49
MoCA	23.82 ± 3.40	22.91 ± 3.75	0.69
FSS	51.36 ± 10.58	51.73 ± 8.92	0.28
HAMA	16.82 ± 10.83	14.27 ± 5.95	0.09
HAMD	15.27 ± 7.17	12.45 ± 5.24	0.25
GDS	17.09 ± 7.09	16.45 ± 6.31	0.53
ESS	7.72 ± 6.96	5.18 ± 3.49	0.23
RBDQ-HK	25.27 ± 13.30	23.18 ± 15.52	0.57
ADL	27.27 ± 11.74	33.18 ± 11.96	0.71

*Continuous variables are represented by Means and standard deviations. ADL, activities of daily living; ESS, Epworth Sleepiness Scales; FSS, Fatigue Severity Scale; HAMA, Hamilton Anxiety Scale; HAMD, Hamilton Depression Scale; H–Y stage, Hoehn and Yahr stage; LEDD, levodopa-equivalent daily dose; MMSE, Mini-Mental State Examination; MoCA, Montreal Cognitive Assessment; MSA-C, Cerebellar subtype of Multiple system atrophy; MSA-P, Parkinsonism subtype of Multiple system atrophy; UMSARS I, Unified Multiple-System Atrophy Rating Scale Part I: historical; UMSARS S II, Unified Multiple-System Atrophy Rating Scale Part II: motor examination; UMSARS IV, Unified Multiple-System Atrophy Rating Scale Part IV: global disability scale; RBDQ-HK, Rapid-eye-movement Sleep Behavior Disorder Questionnaire HongKong*.

### Clinical Efficacy: Primary Outcome

**Figure 1:**
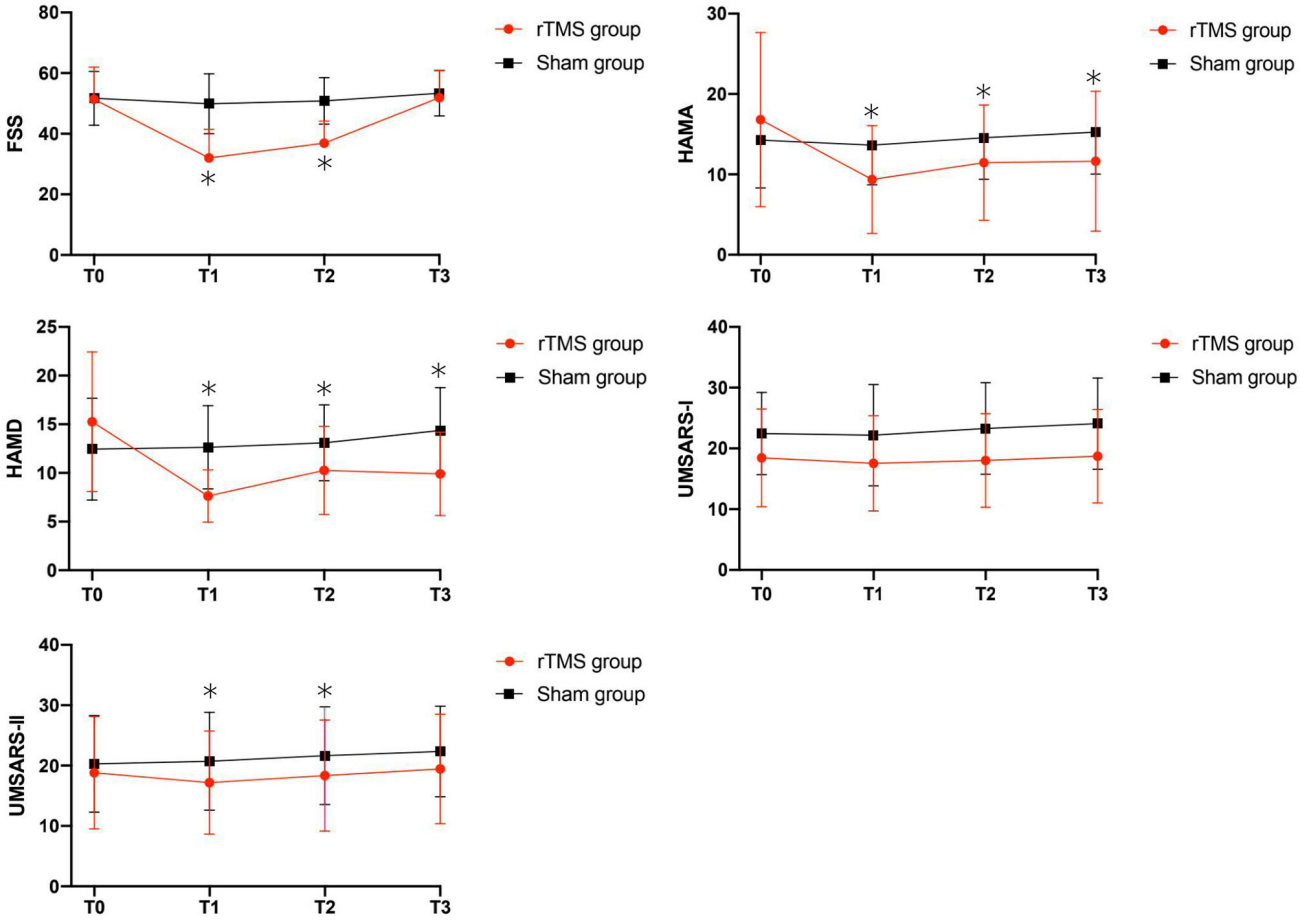
Clinical score changes after the rTMS, including FSS, HAMA, HAMD, UMSARS-I and UMSARS-II scores. Red, rTMS group; black, sham group. *Post-hoc analysis shows significant difference as compared to the baseline (T0) in the group.

As shown in Table 2 and Figure 1, in the comparison of FSS score, there was a significant Group × Time interaction (*p* < 0.01), as well as significant Group (*p* = 0.02), and Time (*p* < 0.001) main effects. *Post-hoc* analyses showed that compared to T0, the rTMS group exhibited remarkable improvements in FSS-9 scores at T1 and T2, but not at T3. No significant improvement was found in the sham group.

**Table 2:** Clinical efficiency of the rTMS and Sham group.

	**rTMS group**	**Sham group**		***DF***	***F***	***P***
**FSS**						
T0 T1 T2 T3	51.36 ± 10.58 32.00 ± 9.45 36.91 ± 7.27 51.91 ± 9.05	51.73 ± 8.92 49.91 ± 9.85 50.82 ± 7.65 53.36 ± 7.46	Group Time Group × time	1 1 1	6.13 36.34 21.53	0.02 <0.001 <0.001
**HAMA**						
T0 T1 T2 T3	16.82 ± 10.83 9.36 ± 6.71 11.46 ± 7.17 11.64 ± 8.71	14.27 ± 5.95 13.64 ± 4.93 14.55 ± 5.17 15.27 ± 5.24	Group Time Group × time	1 1.60 1.60	0.56 7.09 6.28	0.46 0.01 0.01
**HAMD**						
T0 T1 T2 T3	15.27 ± 7.17 7.64 ± 2.69 10.27 ± 4.54 9.91 ± 4.28	12.45 ± 5.24 12.64 ± 4.27 13.09 ± 3.89 14.36 ± 4.39	Group Time Group × time	1 1.92 1.92	2.34 4.29 5.83	0.14 0.02 0.01
**UMSARS-I**						
T0 T1 T2 T3	18.45 ± 8.03 17.55 ± 7.83 18.00 ± 7.71 18.73 ± 7.70	22.45 ± 6.76 22.18 ± 8.32 23.27 ± 7.55 24.09 ± 7.52	Group Time Group × time	1 1.49 1.49	2.22 4.58 1.13	0.15 0.03 0.32
**UMSARS-II**						
T0 T1 T2 T3	18.82 ± 9.32 17.18 ± 8.55 18.36 ± 9.20 19.45 ± 9.08	20.27 ± 8.01 20.73 ± 8.10 21.64 ± 8.10 22.36 ± 8.43	Group Time Group × time	1 1.84 1.84	0.59 10.48 3.43	0.45 <0.001 0.05

*Continuous variables are represented by Means and standard deviations. FSS, Fatigue Severity Scale; HAMA, Hamilton Anxiety Scale; HAMD, Hamilton Depression Scale; UMSARS I, Unified Multiple-System Atrophy Rating Scale Part I: historical; UMSARS S II, Unified Multiple-System Atrophy Rating Scale Part II: motor examination*.

### Clinical Efficacy: Secondary Outcomes

Our analyses revealed significant Group × Time interactions for HAMA scores (*P* = 0.01), HAMD scores (*P* = 0.01), and UMSARS-II scores (*P* = 0.05), indicating that rTMS yielded improvements in these scores compared to sham stimulation. *Post-hoc* analyses showed that compared to T0, HAMA, and HAMD scores were significantly reduced in rTMS group at T1, T2, and T3; while the UMSARS-II scores were significantly improved at T1 and T2, but not at T3. We did not find any significant Group × Time interaction in the comparison of UMSARS-I scores.

### Adverse Events

Few transient and minor adverse events were reported during the stimulation sessions only. Two patients in the rTMS group reported mild and transient headaches after the first session, which lasted around 10 min; while one patient in the sham group reported minor dizziness after the first stimulation.

## Discussion

In this randomized, double-blind, sham-controlled study, we demonstrated that high-frequency rTMS over the left DLPFC induced a short-lasting improvement in fatigue in patients with MSA. In addition, patients' motor symptoms, as well as depression and anxiety symptoms, were also shortly improved to a certain extent after the active rTMS. Though the beneficial effects lasted no more than 4 weeks, we suggest that the high-frequency rTMS over the left DLPFC could still be used as an available therapeutic protocol for alleviating fatigue in patients with MSA.

The beneficial effects of rTMS on fatigue have been previously reported in several neurological disorders, such as fibromyalgia, myalgic encephalomyelitis, and multiple sclerosis, as fatigue is a common symptom in these disorders and contributes substantially to decrements in quality of life and disability (15). A randomized-controlled trial provided evidence that 4 weeks of daily high-frequency (10 Hz) rTMS over the left DLPFC rTMS is able to improve fatigue in patients with fibromyalgia, and provided impetus that the utility rTMS may be an available approach for the relief of fatigue in related disorders (11). Another similar study investigating rTMS effect in patients with fibromyalgia revealed significant improvements in fatigue, depression, and quality of life in the rTMS treatment group when accepting daily high-frequency (10 Hz) rTMS to the left DLPFC over 3 weeks (12). Recently, Yang et al. (13) reported that rTMS over the left DLPFC improves fatigue in patients with myalgic encephalomyelitis and suggested that rTMS can be a novel therapeutic intervention for fatigue. None of the patients previously experienced serious side effects in these studies, which provide compelling evidence for the safety of rTMS treatment. These studies suggest that 10 Hz rTMS over the left DLPFC may be an effective and safe strategy to relieve fatigue in patients with chronic neurological disorders. In the present study, similarly, our findings also provided evidence for the beneficial effects of rTMS, indicating that high-frequency rTMS over the left DLPFC may be a safe and effective therapy for alleviating fatigue in patients with MSA.

It is well-known that the most common and widely used site for rTMS relieving depression is DLPFC (15–18). High-frequency rTMS over the left DLPFC has been suggested as Level A evidence (definite efficacy) for relieving depression, and as Level B evidence (probable efficacy) for improving Parkinson's disease related depression (19). Though fatigue and depression have some clinical features in common, fatigue is distinguishable from depression and indeed an independent entity from depression (20). Fatigue refers to subjective sensations of weariness, increasing sense of effort, mismatch between effort expended and actual performance, or exhaustion (20). However, fatigue and depression, both of which are common non-motor symptoms in patients with MSA, do have similar pathophysiological mechanisms, including serotonergic dysfunction in basal ganglia and limbic circuits, which contribute to dysfunction of prefrontal-basal ganglia loops and impaired integration of limbic input and motor functions (6). Although no evidence-based guidelines have been proposed on the therapeutic use of rTMS for fatigue yet (19), the left DLPFC has been chosen as the optimal stimulation target for fatigue treatment in several previous studies (11–13). Here, we used the same stimulation target and demonstrated similar favorable results. It has been generally suggested that rTMS can not only generate biological effects on the stimulation site per se, but also on other distant sites connected by the activated networks (21). High-frequency rTMS generates a remarkable change of blood-oxygen-level-dependent (BOLD) signal within large and distant areas of the cortex (22). Several studies have also proved that high-frequency rTMS of the DLPFC can increase dopamine release within basal ganglia (23, 24). Furthermore, the effects of high-frequency rTMS might be the result of not only a direct enhancement of motor cortex excitability (25), but also a decrease of inhibitory γ-aminobutyric acid (GABA) neurotransmission-mediated intracortical inhibition (26). Therefore, high-frequency rTMS over DLPFC is assumed to increase the cortex excitability and dopamine release and modulate cortical plastic, which may impart a beneficial effect on fatigue symptom.

In the present study, though we found that rTMS could improve fatigue in MSA patients, the beneficial effect lasted no more than 4 weeks. Differently, in another two randomized, placebo-controlled trials (11, 12), both studies showed long-term favorable rTMS effects on relieving fatigue in patients with fibromyalgia. Such discrepancy may be attributed to the different parameters used in our study, that is, with an intensity of 100% RMT (vs. 120% RMT in their studies), 1,200 pluses per session (vs. 3,000 pulses) and a total of 2 weeks duration (vs. 4 weeks). More importantly, MSA is characterized by a relentless worsening of motor and non-motor symptoms, with a more rapid progression at the onset (1); whereas fibromyalgia is a mild chronic disorder (11). This indicates that at the time of follow-up, patients may already experience aggravation of motor symptoms and non-motor symptoms, which may also contribute to the inconsistency.

In addition, our results showed that rTMS over left DLPFC could also improve depression and anxiety, as well as motor symptoms in patients with MSA. It is generally known that rTMS over the left DLPFC could improve depression, including Parkinson's disease-related depression (16–18). The improvements in MSA-related depression symptoms observed in our study are consistent with the results of many previous studies. Motor symptom scores were also statistically significant improvement between the two groups. As noted previously, the magnetic stimulus has distant actions, which means it not only activates local inter-neuronal circuits, but also those fibers projecting to distant structures. The distant actions of rTMS were initially demonstrated in some studies, which showed that rTMS over the DLPFC can modulated M1 excitability, even at a higher extent than direct M1 stimulation itself (15, 17). It may provide evidence that motor symptoms were improved through rTMS stimulating over the DLPFC in our study.

Our study has several limitations. First, the study was conducted in a single center and the number of participants is relatively small. Second, as the diagnosis of early MSA is full of challenge, only patients with a clear diagnosis were enrolled in our study, indicating a relatively more sever disease. Future studies with a bigger sample size enrolling more MSA patients in the early stage are warranted to clarify the rTMS effect on fatigue in MSA patients.

## Conclusions

In conclusion, our findings suggest that high-frequency rTMS over left DLPFC may ultimately serve as an add-on therapy for alleviating fatigue in MSA patients, though the beneficial effects last no more than 4 weeks. In the future, more optimistic rTMS protocols and techniques are needed to prolong the treatment effect in routine clinical practice.

## Data Availability Statement

The raw data supporting the conclusions of this article will be made available by the authors, without undue reservation.

## Ethics Statement

The studies involving human participants were reviewed and approved by the Xuanwu Hospital Ethics Committee. The patients/participants provided their written informed consent to participate in this study.

## Author Contributions

PC, J-HM, and HS designed the study. JP carried out data collection, analyzed the data, and drafted the manuscript. PC and T-MM revised the manuscript. All authors have read and approved the final version for publication.

## Funding

This work was supported by grants from the National Key R&D Program of China (Nos. 2018YFC1312001 and 2017YFC0840105), Beijing Municipal Administration of Hospitals' Mission Plan (Code: SML20150803), Beijing Municipal Science and Technology Commission (No. Z171100000117013), and Inner Mongolia Natural Science Foundation (No. 2020MS08138).

## Conflict of Interest

The authors declare that the research was conducted in the absence of any commercial or financial relationships that could be construed as a potential conflict of interest.

## Publisher's Note

All claims expressed in this article are solely those of the authors and do not necessarily represent those of their affiliated organizations, or those of the publisher, the editors and the reviewers. Any product that may be evaluated in this article, or claim that may be made by its manufacturer, is not guaranteed or endorsed by the publisher.
